# Parental Education and Pre-School Children’s Objectively Measured Sedentary Time: The Role of Co-Participation in Physical Activity

**DOI:** 10.3390/ijerph15020366

**Published:** 2018-02-20

**Authors:** Suvi Määttä, Carola Ray, Henna Vepsäläinen, Elviira Lehto, Riikka Kaukonen, Anna Ylönen, Eva Roos

**Affiliations:** 1 Samfundet Folkhälsan, Folkhälsan research Center, 00250 Helsinki, Finland; Carola.ray@folkhalsan.fi (C.R.); Elviira.lehto@folkhalsan.fi (E.L.); Riikka.kaukonen@folkhalsan.fi (R.K.); Anna.ylonen@folkhalsan.fi (A.Y.); Eva.roos@folkhalsan.fi (E.R.); 2 Department of Food and Environmental Sciences, University of Helsinki, 00014 Helsinki, Finland; henna.vepsalainen@helsinki.fi; 3 Department of Public Health, University of Helsinki, Clinicum, 00014 Helsinki, Finland

**Keywords:** children, parents, physical activity, sedentary lifestyle, mediation analysis

## Abstract

Parental co-participation in physical activity (PA) may be a beneficial parenting practice for diminishing children’s sedentary time (ST). Less information is available, however, on the explanatory role of co-participation in PA regarding parental educational differences in children’s ST. Preschool-aged children (N = 864, mean age 4.8, 52% boys) with their parents participated in a cross-sectional DAGIS (Increased Health and Wellbeing in Pre-schools) study between years 2015 and 2016. Children (N = 821) wore an accelerometer for one week. Parents were informed of their educational background, and the frequency of visits with their child in nature, to parks or playgrounds, their own yard, and indoor sport facilities (N = 808). Testing the associations required multiple regression analyses. Parents with a low educational background reported more frequent visits with their child to their own yard, and these visits were associated with children’s lower ST. More highly educated parents co-visited indoor sport facilities more frequently, although this did not have a significant association with children’s ST. More frequent visits in nature were associated with a lower ST at weekdays, regardless of educational background. Future health promotion strategies should inform parents that frequent co-participation in PA, for example, in one’s own yard, is beneficial for lowering children’s ST.

## 1. Introduction

The pre-school years (children roughly aged 3 to 5) are a period of rapid growth and development. During this period, habits begin to shape regarding energy balance-related behaviours (EBRBs), such as dietary intake, physical activity (PA), and sedentary behaviour (SB) [1]. EBRB habits learned at this age have a tracking tendency, predicting EBRBs and health outcomes in adulthood [2,3,4]. Therefore, the pre-school-age is considered a critical age period in the promotion of healthy EBRB habits, alongside others such as recommended levels of PA and low levels of SB [3]. Sedentary behaviour is defined as any waking behaviour characterised by an energy expenditure ≤1.5 metabolic equivalents while in a sitting, reclining, or lying posture [5]. The time spent in different SBs is called sedentary time (ST), usually measured by objective measurements such as accelerometers [6].

Recent studies demonstrate that most pre-school children have high levels of ST, and do not achieve the recommended levels of PA [7,8]. In Finland, at least 3 hours a day of PA at any intensity level, to limit prolonged ST and avoid continuous ST lasting over one hour, is among the current national recommendations for pre-school-aged children [9]. These recommendations are based on studies indicating the diametrically opposed health effects of PA and ST. Regular PA at pre-school age is associated with multiple health benefits [10]. Studies using television viewing as an indicator of ST have shown negative health consequences of extensive television viewing on pre-school children’s health, whereas the health consequences of overall ST among pre-school children are still poorly understood [11,12]. Some recent studies link a higher ST with higher odds of belonging to the metabolically unhealthy group of school-aged children; high ST is also linked with obesity risk markers among school-aged children [13,14]. Studies among adults indicate an association between high levels of ST and negative health consequences, such as type 2 diabetes, all-cause mortality, and cardiovascular disease incidence [15,16,17,18]. Given the tracking tendency, future health promotion strategies must identify how to change behaviours among pre-school-aged children from spending time in ST to being more activity-oriented.

Parents are central to acquainting their children with different EBRB habits through their different parenting styles and practices [19]. As per social-cognitive theory [20], children learn by observing significant others, such as parents, and their habits and behaviour. A child consequently uses this observed knowledge to guide and develop their own habits and behaviour [20]. Co-participation in PA, defined as parents engaging in PA with their child [21], is one form of parenting practice in which a child can observe and learn healthy PA habits together with their own parent. Co-participation in PA allows, not only direct parental role modelling, but also occasions for encouragement and support to develop motor skills and feelings of competence [20]. Several studies among school-aged children argue that frequent co-participation in PA increases children’s PA levels [22,23], although it is also stated that friends and other social environments may be more important to school-aged children’s PA than parents’ participation [24,25]. A greater role of parents may be expected among pre-school-aged children’s daily activity choices due to children’s early developmental stages [26]. The pre-school-aged child that is involved in PA needs parental involvement in reaching the places for the activity, some form of parental support in engaging the activity, and parental presence throughout the activity. Previous studies state that co-participation in PA as a family seems to be greatest during early childhood [24,25]. However, to our knowledge, only one study has specifically studied the influence of co-participation in PA on pre-school children’s activity levels. Hnatiuk [27] discovered most Belgian mothers reported infrequent co-participation in PA with their children. In particular, walking or cycling together was associated with higher PA at weekends in both children and mothers [27]. Other studies have concluded that higher parental PA levels are associated with higher PA levels of pre-school children [28], but have not specified whether the activity was performed together. Some results have indicated parents and children spend most of their time together in SBs [29,30], but have not focused on the frequency of co-participation in PA.

A statistically non-significant overall association is commonly found between parental educational background and pre-school children’s ST [31]. Intervening factors (mediators) can exist, however, between the associations of parental educational background and children’s ST. Current practices recommend conducting mediational analyses despite non-significant overall associations, as this would enable an easier understanding of the mechanisms of influence [32,33]. For instance, school-aged children with high educational family backgrounds are known to have more co-participation in PA than children with low educational family backgrounds [34]. Whether the association between parental educational background and co-participation in PA also influences children’s activity levels is less obvious. As Gubbels et al (2011) [35] theorise, parental education is considered a more distal background factor that influences both parenting practices and children’s behaviours in the model of energy balance-related parenting [35]. Based on this model, we hypothesise that co-participation in PA as a form of parenting practice acts as a mediator in the associations between parental education and children’s ST.

The current study aims to investigate the associations between parental education, co-participation in PA, and pre-school-children’s ST. We set three aims for our study: to determine if 1) educational differences exist in co-participation in PA, that is, the frequency of visiting nature, parks, yards, and indoor sports facilities, 2) co-participation in PA is associated with pre-school children’s ST when adjusted for parental education, and 3) co-participation in PA acts as a mediator in the association between parental education and children’s ST.

## 2. Materials and Methods

### 2.1. Study Design and Participants

The investigation of socioeconomic status (SES) differences in pre-school children’s EBRBs is facilitated by a multi-year research project named DAGIS (Increased Health and Wellbeing in Pre-schools). This research project involved a cross-sectional study, conducted between autumn 2015 and spring 2016. The cross-sectional study focused on recognising the possible SES differences in pre-school children’s EBRBs and discovering the factors that explained these differences. The study participants were children that were attending pre-schools aged 3 to 6 years, their parents, and pre-school staff. The study involved a total of 8 municipalities and their pre-schools in southern and south-west Finland. The participating pre-schools in municipalities were selected randomly, covering both urban and rural environments.

The recruitment of parents of 3 to 6-year-old pre-school children was implemented through pre-schools by providing an information letter with a consent form. Written consent was provided by a total of 983 families (27% of invited). Final participation in the study totalled 24% of children (N = 864) from 66 pre-schools. The study was conducted in accordance with the Declaration of Helsinki. The University of Helsinki Ethical Review Board in the Humanities and Social and Behavioural Sciences approved the study procedures (the ethical statement number 6/2015).

### 2.2. Children’s Sedentary Time

Children wore Actigraph wGT3X-BT (Pensacola, Florida, USA) accelerometers for 7 consecutive days, 24 hours per day. An actigraph is a valid and reliable measure of children’s ST and PA, and is extensively used for the objective measurement of these behaviours among children [36,37,38]. Research assistants set an accelerometer on children’s waists in pre-school, and the accelerometer was collected from pre-schools after the data collection period. During the period of accelerometer use, parents reported in a diary their child’s pre-school hours and possible non-wearing hours.

When downloading data from the Actigraphs, we chose a 15-s-epoch length [39]. Non-wearing time was set at 10 min or more consecutive zeros. During the formation of ST variables (0–25 counts/15 s), we applied Evenson cut-points [40] because these cut-points often give the best ST classification accuracy for children of all ages [36,41,42].

This study involved forming two different variables: weekday ST and weekend ST. Reported sick-days, hours in pre-school, weekdays when a child was absent from pre-school, and night sleeping-hours were excluded when forming the variables. Children’s possible nap times were not excluded from the data. Weekend and weekday ST were analysed separately, because we expected possible parental co-participation in PA to be greater at weekends than on weekdays. Greater co-participation would consequently more strongly influence children’s ST. All the children in our study attended pre-school. Weekday ST consisted of waking hours before and after pre-school on at least two days during the measurement week. Only the weekdays when child had been in preschool were included in the weekday ST variable. On the selected weekdays, children had been at least 360 min in pre-school per day. Weekend ST was calculated if both days contained at least 600 min of data per day. To illustrate the average ST minutes in one hour, we divided both weekend and weekday ST variables by the wearing hours and multiplied by 60 min.

### 2.3. Parental Education

The consent form assessed the educational background of parents. Parents choose their highest educational background from a ready-made seven-item list. The answers were re-grouped into three categories: low = high school or vocational school graduate or lower, medium = Bachelor’s degree or equivalent, and high = Master’s Degree or higher.

### 2.4. Co-Participation in PA

One parent of the family completed the questionnaire, either in paper- or electronic form. As recommended in a recent review and previously used in this age-group, co-participation in PA was measured by capturing the frequency of co-participation and type of activities completed [27,43]. The type of activities included in the question were purposefully designed for the Finnish context based on our formative work among Finnish parents with preschool-aged children [44]. In the questionnaire, parents reported how often their child goes to the following places with at least one adult in the family: nature or forest, parks and playgrounds, and own yard and indoor sport facilities, such as swimming pools. The answer options for each place were: less than once a month, 1–3 times per month, 1–2 times per week, 3–4 times per week, 5–6 times per week, and daily. The original answer options were recoded to distinguish the most frequent visitors from the others. The frequency of visits in nature and forests was recoded so that answer options from ‘1–2 times per week’ to ‘daily’ were combined to mean often (all other values = seldom). Frequency of visits to parks and playgrounds was similarly dichotomised. The frequency of visits to one’s own yard was recategorised into three groups, so that the frequency of the highest category was daily, of the middle category was three to six times a week, and of the lowest group was between one and two times a week and less than once a month. The frequency of visits to an indoor sport facility was also categorised into three groups: the highest group consisted of visits at least once a week, the middle group consisted of visits of 1 to 3 times per month, and the lowest group represented frequency of visits less than once a month.

### 2.5. Covariates

Covariates in the mediation analyses comprised a guardian’s relation to the child (mother or father), a child’s age and gender, and season of measurement. Guardians other than mothers and fathers (for example, grandparents) who had filled in the parental questionnaire (N = 4) were excluded. The season of measurement covered three categories: early autumn (September–October), late autumn (November–December), and spring (January–April).

### 2.6. Statistical Analyses

Applying SPSS statistical program (IBM *SPSS* Statistics: Chigaco, IL, USA) allowed checking of the descriptive statistics and spearman correlations. We removed the outliers over 3 standard deviations (N = 1 in weekday ST).

Conduction of the main analyses involved the latent variable modeling program Mplus 7.11 (Muthén & Muthén, Los Angeles, CA, USA). The analyses used the following steps: (a) associations between parental education and co-participation in PA (a-paths), (b) associations between co-participation in PA and children’s ST when adjusted with parental education (b-paths), and (c) indirect associations (a*b) between parental education and children’s ST through each co-participation variable. A statistically significant indirect effect existed if the confidence interval did not include zero, employing 95% bias corrected confidence intervals. Adjustment of confidence intervals in the mediation models related to clustering at the family level, that is, nested design of participating siblings from a family. The highest educational background was treated as the reference group, applying maximum likelihood estimation with robust standard errors (MLR) as an estimator.

## 3. Results

A total of 822 children, aged 3 to 6, wore an accelerometer for one week. Of these children, 778 (47.5% girls) had valid data from home time in pre-school days, and 779 (51.5% girls) had valid data from weekends. A total of 808 parents completed the parental questionnaire. Of the respondents, 12% (N = 95) were fathers. The mean age of parents was 35.9 years (standard deviation 4.89). Table 1 presents the detailed descriptive statistics.

Parental educational background correlated negatively with frequency of visits to own yard (r = −0.105) and positively with frequency of visits to an indoor sport facility (r = 0.159). Children’s home ST during pre-school days and children’s weekend ST correlated negatively with frequency of visits to own yard (r = −0.152 and r = −0.134, respectively). Table 2 presents the Spearman correlations.

### 3.1. Associations between Parental Education and Co-participation in PA (a-paths)

Table 3 presents the results of associations between parental education and co-participation in PA (a-paths). On the one hand, parents with low parental educational backgrounds reported more frequent visits with their child to their own yard than parents with high parental educational backgrounds. On the other hand, parents with high parental educational backgrounds reported more frequent visits to indoor sports facilities than parents with low or middle educational backgrounds.

### 3.2. Associations between Co-participation in PA and Children’s ST (b-paths)

Table 4 presents the results of associations between co-participation in PA and children’s ST when adjusted for parental educational background (b-paths). More frequent visits in nature were associated with lower children’s weekday ST. More frequent visits to own yard were associated with lower children’s ST, both on weekdays and at weekends.

### 3.3. Indirect Associations of Potential Mediators in Associations between Parental Education and Children’s ST (indirect effect, a*b)

Table 5 presents the indirect effect of each potential mediator in the associations between parental education and children’s ST. Children with low parental educational background visited in their own yard with their parents more frequently, and this was associated with lower ST on weekdays and at weekends compared to children with high parental educational background.

## 4. Discussion

Our study found that parents with a low educational background reported more frequent visits with their child to their own yard, and this frequency was associated with children’s lower ST both during weekdays and at weekends. In addition, parents with high educational backgrounds reported more frequent visits with their children to indoor sports facilities. Frequent visits in nature were associated with lower children’s weekday ST.

Pre-school-aged children are heavily dependent on their parents to create opportunities for structured play and active plays, but also for SBs [26]. Co-participation in PA may have multiple benefits for children in learning healthy habitual behaviour. Children learn habits by observing their parents, and accordingly develop their own habits and behaviour, as per socio-cognitive theory [20]. Our study suggests that co-participation in PA near the home, that is the yard and nature, is especially associated with a lower children’s ST. Other studies have mentioned a double fold effect of unstructured play, such as the type of play engaged in by pre-school children in nature and yard. Unstructured play can both increase pre-school-aged children’s PA [45,46] and diminish children’s ST [47]. Multiple reasons exist to explain how this type of co-participation in PA is significantly associated with children’s ST. Other studies suggest that environmental greenness, overall time spent outdoors, or parental involvement are associated with higher pre-school children’s moderate-to-vigorous PA [26,48,49,50]. Co-participation in PA in own yard and nature combine all these aspects. Our study also suggests that this type of co-participation is beneficial for diminishing pre-school children’s ST. The role of one’s own yard for co-participation in PA should be accentuated. Parents with low educational background reported more frequent visits to their yard, and this frequency was also associated with lower children’s ST. A review by Maitland et al (2013) noticed that studies investigating the role of one’s own yard on ST and PA levels are scarce. They recommended further studying the role of the yard on children’s ST, independent of PA [51]. Our study highlights the need for additional knowledge on the importance of ones’ own yard and its associations with children’s ST.

Unstructured play near the home may especially influence children with low parental educational backgrounds. Two recent studies support our findings. One study of 6 to 8-year-old Finnish children concluded that children with a lower parental SES background (both educational and income background) had more unstructured type of PA such as playing outdoors unsupervised compared to children with a higher SES background, who often partake in organised sports [52]. Another study among low-income parents stated that frequent park use with their pre-school child nearby the home reduced ST for both child and parent [47]. Overall, our study suggests that parental educational background is associated with particular forms of co-participation in PA. A lower educational background was associated with more frequent visits to own yard, whereas a higher educational background was associated with more frequent visits to indoor facilities; frequent visits to indoor facilities were not associated with children’s ST. Similarly, Hnatiuk et al (2017) found that visits to indoor facilities at weekends were associated with children’s lower moderate-to-vigorous PA levels [27]. However, visits to indoor facilities often require travelling by car or public transportation; this seated travel time may reverse for the possible PA gained in indoor facilities.

No constant information exists regarding the association between parental educational background and pre-school-aged children’s ST [31]. Our study also failed to find significant associations between parental educational background and children’s ST [53]. A better understanding of the potential mechanisms influencing children’s behaviour requires mediational analyses. Given the tracking tendency of SB and PA [3], developing strategies in the early years is useful in preventing possible parental educational differences in ST. Various interventions focusing on co-participation in PA have successfully increased PA among school-aged children [54]. Future intervention strategies and health promotions projects could underline the importance of co-participation in PA, and create family habits around this behaviour to allow co-participation becoming habitual for both children and parents. Useful advice for parents involves highlighting the value of unstructured play in a yard to diminish pre-school children’s ST. Therefore, co-participating in visits to expensive indoor sport facilities is not necessary to activate a pre-school child, whereas frequent visits to ones’ own yard and near the home are beneficial in decreasing children’s ST.

The role of co-participation in PA requires further exploration, because parents’ actual influence on children’s activity levels is unclear. A recent study by Sleddens (2017) [55] concluded that parents and children mutually influence each other’s behaviour. This study recommends further analysis of the bi-directional parent-child interactions in the stimulation of PA [55]. Co-participation in PA can decrease parents’ activity levels [27], although a recent study highlighted the association between co-participation in PA and parents’ lower ST [47]. These contradictory findings may be explained by the form of co-participation studied, that is, visits to own yard or indoor sports facilities. Instead of being active together with their child, a parent might supervise in sitting or standing positions. A study in a pre-school setting [56] speculated that early educators’ supervision of outdoor recess was associated with lower moderate-to-vigorous PA levels among pre-school girls. Depending on the type of co-participation, parents may similarly influence children’s activity. The activity levels of parents participating in our study are unknown, and we did not ask what the parents themselves do when co-participating in PA with their child. This area is, therefore, a possible topic for future studies. Similarly, the role of co-participation in PA may be different for boys than girls. Future studies could study, for instance, whether maternal co-participation is more beneficial to girls than boys. Children’s age may also influence the frequency of PA co-participation. Future studies could separately study children of different age groups. We selected four different places in which co-participation in PA is possible. Although these places were selected as particularly suitable to the Finnish context, other places also allow co-participation in PA. However, our co-participation question regarding PA covered both frequency of co-participation and type of activities done together, as recommended in a recent review [43]. If study logistics had allowed, objective measurements, such as Bluetooth and GPS devices, would have provided more extensive knowledge about the nature of co-participation in PA. As discussed in recent review, the operationalisation of co-participation in PA needs more comprehensive, consistent, and validated overall measurement that captures existence, frequency, duration, and/or type of co-participation and social aspects of co-participation [43].

Our study had certain limitations. Differentiating between lying down or sitting from other very light intensity activities, such as standing still, is difficult with hip-placed accelerometers. Movements among pre-school-aged children are naturally sporadic, and children are rarely in standing positions for long periods of time. The indirect effect sizes are generally small, but specific indirect effects in mediator models are usually attenuated to the extent that the mediators are correlated [33]. The low participation rate in our study may influence the generalisability of our findings. We also acknowledge that most of our participants were from two-parent families, and co-participation in PA may be different in single-parent families. In addition, shift work may influence co-participation during PA time. Similarly, other SES indicators (such as income) may produce different results. Therefore, more research is needed to confirm our findings. The cross-sectional nature of our study limits the ease of identifying the direction of associations between co-participation in PA and children’s ST. We acknowledge that fathers’ co-participation in PA may be different than mothers’. The formulation of our question referred to at least one parent participating with a child; therefore, we did not expect the respondent to also be the co-participation participant. Although most of our participating children attended at least four days per week in pre-school, the children with less than four-days-attendance may have more frequent possibility to co-participate in PA during weekdays with their parents. We did not measure the distance to different PA places in our parental questionnaire. In Finnish context, the distance to indoor sport facilities in particular may be dependent on the context (e.g., rural/urban) that the family is living in. Future studies could study more profoundly how the rural or urban setting influences in co-participation in PA.

Our study has many strengths: we highlighted the effect of variations in parental educational background on different forms of co-participations in PA, and how co-participation in PA is associated with children ST. Mediational analyses introduced new knowledge of potential mechanisms of influence regarding parental educational background differences and children’s ST. Other important issues relate to the objective measurement of children’s ST. By separating weekend ST and home ST during pre-school days, we could separately study factors associated with ST. We focused on various potential places of co-participation allowing PA. Our sample consisted of almost 800 children aged 3 to 6, from different municipalities and environmental settings, including both rural and city surroundings. Based on our study strengths, we presented unique information about the role of co-participation in PA on pre-school children’s ST.

## 5. Conclusions

In conclusion, our study identified that parents with low educational backgrounds reported more frequent visits with their child to their own yard. This type of co-participation was associated with children’s lower ST than children with high parental educational backgrounds. Accordingly, parents with a high educational background reported more frequent visits to indoor sport facilities, and frequent visits in nature were associated with children’s lower ST regardless of parental educational background. Our observations suggest that future health promotion strategies could develop the knowledge of the importance of PA co-participation in diminishing pre-school children’s ST. Parents should be informed that co-participation in PA in the form of unstructured play in yards and nature near the home is especially valuable in diminishing children’s ST. These unstructured forms of co-participation in PA are also widely accessible formats of co-participation in PA for parents of all educational backgrounds.

## Figures and Tables

**Table 1:** Descriptive statistics of variables used in Increased Health and Well-being in Preschool (DAGIS) study.

Measures		Descriptives in % If not Stated Otherwise (N)
**Children**		
Age		Mean 4.75 (standard deviation 0.89)
	3 years	20.6
	4 years	36.7
	5 years	35.0
	6 years	7.6
Gender, % boys		52 (N = 450)
Participating siblings in the DAGIS study		11
Type of household child is living in		
	Two-parent family	91.7 (N = 737)
	Single-parent family	5.8 (N = 47)
	Other	2.5 (N = 35)
Preschool attendance		
	5 days per week	64.5 (N = 515)
	4 days per week	18.1 (N = 145)
	1–3 days per week	17.4 (N = 141)
Weekday sedentary time		Mean 29.76 min (standard deviation 4.99) per hour
Weekend sedentary time		Mean 28.47 (standard deviation 4.76) per hour
Measurement season		
Season of accelerometer wearing	September–October	43.4 (N = 375)
	November–December	36.0 (N = 311)
	January–April	20.6 (N = 178)
**Parents**		
Parental educational background		
	Low	29.3 (N = 232)
	Middle	41.3 (N = 327)
	High	29.4 (N = 233)
Frequency of co-participation in PA		
Frequency of visits in nature	Seldom	56 (N = 444)
	At least once a week	44 (N = 353)
Frequency of visits to parks and playgrounds		
	Seldom	48 (N = 382)
	At least once a week	52 (N = 416)
Frequency of visits to own yard		
	Seldom	25 (N = 196)
	Often	40 (N = 317)
	Daily	35 (N = 281)
Frequency of visits to an indoor sports facility (e.g., swimming pools)		
	Seldom	26 (N = 205)
	1–3 times a month	34 (N = 268)
	Every week	41 (N = 325)

**Table 2:** Spearman correlations between the measures used in the study (listwise N = 695).

Variable	1.	2.	3.	4.	5.	6.
1.Parental educational background						
2. Children’s weekday sedentary time ^1^	−0.004					
3. Children’s weekend sedentary time ^1^	0.018	0.649 ***				
4. Frequency of visits in nature	−0.050	−0.035	−0.039			
5. Frequency of visits to parks and playgrounds	0.002	−0.060	−0.003	0.193 ***		
6. Frequency of visits to own yard	−0.105 *	−0.152 ***	−0.134 ***	0.220 ***	0.078 *	
7. Frequency of visits to an indoor sports facility	0.159 ***	−0.005	0.052	0.049	0.091 *	0.042

* *p* < 0.05, ** *p* < 0.01, *** *p* < 0.001; ^1^ average minutes in one hour.

**Table 3:** The associations between parental educational background and co-participation in physical activity adjusted for gender of parental questionnaire respondent, measurement season, and child’s gender and age *.

Parental Educational Background	Frequency of Visits in Nature (N = 788)			Frequency of Visits to Parks and Playground (N = 789)			Frequency of Visits to Own Yard (N = 789)			Frequency of Visits to an Indoor Sports Facility (N = 789)		
	β	Lower 95% CI	Upper 95% CI	β	Lower 95% CI	Upper 95% CI	β	Lower 95% CI	Upper 95% CI	β	Lower 95% CI	Upper 95% CI
Low	0.07	−0.03	0.17	0.01	−0.10	0.10	**0.21**	**0.05**	**0.36**	−**0.32**	−**0.48**	−**0.16**
Middle	0.02	−0.07	0.11	−0.04	−0.13	0.05	0.12	−0.02	0.26	−**0.15**	−**0.29**	−**0.02**
High (reference)												

CI = confidence interval; *statistically significant associations in bold.

**Table 4:** The associations between co-participation in physical activity and children’s sedentary time (ST) on weekdays (N = 789) or on weekends (N = 786), adjusted for gender of parental questionnaire respondent, child’s gender and age, measurement season, and parental educational background *.

Co-participation in PA	Children’s Weekday ST			Children’s Weekend ST		
	β	Lower 95% CI	Upper 95% CI	β	Lower 95% CI	Upper 95% CI
Frequency of visits in nature (N=788)	**−0.74**	**−1.42**	**−0.06**	−0.63	−1.26	0.01
Frequency of visits in parks and playground (N = 789)	−0.55	−1.24	0.14	−0.16	−0.80	0.49
Frequency of visits in own yard (N = 789)	**−0.77**	**−1.22**	**−0.31**	**−0.70**	**−1.13**	**−0.27**
Frequency of visits in an indoor sports facility (N = 789)	−0.15	−0.56	0.28	0.14	−0.26	0.55

CI = confidence interval; * statistically significant associations in bold.

**Table 5:** Indirect effects of potential mediators in the associations between parental educational background and preschool children’s sedentary time (ST) on weekdays (N = 789) and on weekends (N = 786) adjusted for gender of parental questionnaire respondent, child’s gender and age, measurement season, and parental educational background *.

Independent Mediation Effect of the Potential Mediators	Parental Educational Background	Children’s Weekday ST			Children’s Weekend ST		
		β	Lower 95% CI	Upper 95% CI	β	Lower 95% CI	Upper 95% CI
Frequency of visits in nature (N = 787)	Low	−0.05	−0.14	0.03	−0.04	−0.12	0.03
	Middle	−0.01	−0.08	0.05	−0.01	−0.07	0.05
	High (reference)						
Frequency of visits in parks and playground (N = 788)	Low	−0.01	−0.06	0.05	0.01	−0.02	0.02
	Middle	0.02	−0.04	0.08	0.01	−0.02	0.04
	High (reference)						
Frequency of visits in own yard (N = 788)	Low	**−0.16**	**−0.31**	**−0.01**	**−0.14**	**−0.29**	**−0.01**
	Middle	−0.09	−0.20	0.03	−0.09	−0.20	0.03
	High (reference)						
Frequency of visits in an indoor sports facility (N = 788)	Low	0.05	−0.09	0.19	−0.05	−0.18	0.09
	Middle	0.02	−0.05	0.09	−0.02	−0.08	0.04
	High (reference)						

CI = confidence interval; * statistically significant associations in bold.
